# Recent Advances in 10-Hydroxy-2-Decenoic Acid (10-HDA): Biosynthesis, Biological Functions, and Regulatory Mechanisms in Honeybees

**DOI:** 10.3390/foods15142458

**Published:** 2026-07-10

**Authors:** Peiyuan Zou, Yunxiao Hu, Bin Yuan, Pengbo Liang, Shanshan Li, Fuliang Hu

**Affiliations:** College of Animal Science, Zhejiang University, Hangzhou 310058, China; zoupeiyuan2216@163.com (P.Z.); huyunxiao1115@163.com (Y.H.); yuan_bin322@zju.edu.cn (B.Y.); pbliang@zju.edu.cn (P.L.)

**Keywords:** 10-hydroxy-2-decenoic acid (10-HDA), mandibular gland, biosynthetic pathway, molecular regulation, functional fatty acid

## Abstract

Royal jelly (RJ) is a highly valued bee-derived functional food and natural health product, in which 10-hydroxy-2-decenoic acid (10-HDA) represents the most characteristic lipid component. As a unique fatty acid found exclusively in RJ, 10-HDA serves not only as a key marker for product authenticity, freshness, and quality evaluation but also as a major contributor to the biological activities of RJ, including immunomodulatory, metabolic regulatory, antimicrobial, anti-inflammatory, antitumor, and dermatological effects. Given its nutritional and quality-related importance, and because most previous reviews have focused primarily on the biological activities or compositional characteristics of 10-HDA, current knowledge regarding its biosynthesis, secretion, and regulatory mechanisms in worker mandibular glands has not yet been systematically organized and summarized. Understanding these processes is essential for explaining the biological origin of 10-HDA accumulation in RJ and for developing strategies to improve 10-HDA yield, royal jelly quality, and production standardization. This review summarizes current knowledge on the physicochemical properties and health-related functions of 10-HDA and further integrates recent advances in its endogenous biosynthesis and regulatory mechanisms. Particular emphasis is placed on the proposed three-step biosynthetic pathway, beginning with stearic acid and proceeding through cytochrome P450-mediated ω-hydroxylation, successive β-oxidation, and terminal dehydrogenation. We also discuss how 10-HDA production is shaped by worker developmental stage, glandular maturation, genetic background, dietary nutrients, botanical origin, endocrine signals, and apicultural management practices. By linking the biological origin of 10-HDA with its functional properties and quality-determining role in RJ, this review provides an integrated framework for understanding the formation of 10-HDA-rich royal jelly. By linking the biological origin, functional properties, and quality-determining role of 10-HDA in RJ, this review provides an integrated framework for understanding 10-HDA-rich royal jelly. It also identifies key gaps in biosynthetic validation, secretion mechanisms, and regulatory networks, offering guidance for RJ quality standardization, production optimization, and functional food development.

## 1. Introduction

Royal jelly is a complex secretion produced by the hypopharyngeal and mandibular glands of worker bees, serving as the essential nutritional substance that enables the exceptional longevity and fecundity of the honeybee queen (*Apis mellifera*) [1]. Within its diverse chemical makeup, 10-HDA has become a key focus of biochemical research due to its unique molecular structure and strong biological effects [2]. As 10-HDA is a unique active substance in RJ, 10-HDA is used as one of the most widely accepted quality markers [3]. Experimental studies have suggested potential biological activities of 10-HDA, especially in maintaining metabolic balance and supporting immune health; however, the biochemical pathway of its endogenous production remains complex. The transition from precursor conversion to final secretion involves intricate metabolic processes that are not yet fully understood [4]. By reviewing recent advancements in biochemical pathways and molecular regulation, this article provides a clear overview of the 10-HDA biosynthetic process. Additionally, we analyze the key internal and external factors influencing its production, from genetic background to environmental conditions, thereby providing an updated overview of current knowledge and identifying future research directions for high-quality apicultural production and the biotechnological use of this bioactive lipid.

## 2. Physicochemical Properties and Biosynthetic Pathways of 10-HDA

10-HDA, colloquially known as “Queen bee acid,” is the predominant lipid synthesized by honeybee mandibular glands. As a signature bioactive organic acid, 10-HDA accounts for more than 1.4% of RJ’s dry weight [5,6]. Structurally, it is defined as a straight-chain unsaturated fatty acid with the formula HO-(CH_2_)_7_CH=CHCOOH [7]. Due to its chemical stability and pleiotropic pharmacological effects, 10-HDA is recognized as the gold-standard physicochemical marker for RJ quality grading. Beyond its role as a quality indicator, 10-HDA exerts broad-spectrum antimicrobial activity against various bacteria and fungi, serving as a natural preservative to maintain microbial inhibition [5,8]. Although the biosynthetic pathway of 10-HDA requires further research to be fully elucidated, current evidence already provides a plausible outline of its synthesis. Stable isotope-labeling studies demonstrated that worker mandibular glands can synthesize mandibular fatty acids from acetate and use octadecanoic acid/stearic acid as a major precursor. In this model, stearic acid is first hydroxylated at the ω-position to form an 18-carbon ω-hydroxy fatty acid, which is then shortened through β-oxidation to generate 10-carbon ω-hydroxy intermediates, including 10-hydroxydecanoic acid (10-HDAA) [7,9]. These steps are supported by tracer experiments in honeybee mandibular glands, whereas the specific enzymes responsible for each reaction remain incompletely characterized. Proteomic studies further showed that fatty acid synthase, long-chain fatty acid-CoA ligase, electron transfer flavoprotein β (ETF-β), and 3-ketoacyl-CoA thiolase are enriched in mandibular glands with high 10-HDA production, suggesting the involvement of fatty acid synthesis and β-oxidation-related enzymes [5,10]. Among these candidates, ETF-β has functional support from RNAi experiments, as its knockdown reduced 10-HDA production in worker mandibular glands [5]. However, other enzymes in this pathway are largely inferred from proteomic or transcriptomic associations and still require direct biochemical validation.

**Figure 1:**
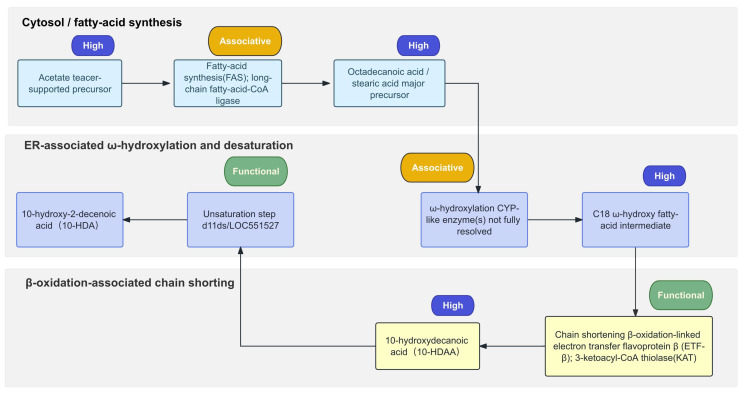
Schematic summary of the proposed 10-HDA biosynthetic pathway in worker honeybee mandibular glands. The figure integrates precursor molecules, candidate enzymes, putative cellular compartments, and the relative level of supporting evidence. Stable isotope studies support acetate and stearic acid as precursors and a chain-shortening model; proteomic and transcriptomic studies identify associated enzymes and pathway components; RNAi experiments provide functional support for selected candidates involved in β-oxidation-associated chain shortening, such as ETF-beta and 3-ketoacyl-CoA thiolase (KAT), and for d11ds/LOC551527, an acyl-CoA Δ11 desaturase-like homolog associated with the unsaturation step.

The enzymatic step responsible for introducing the trans double bond should be interpreted with particular caution. Earlier pathway descriptions often proposed that the double bond is introduced into the shortened ω-hydroxy fatty acid chain by a desaturase [5,7]. In microbial whole-cell systems, acyl-CoA dehydrogenase-related enzymes such as FadE, together with acyl-CoA synthetase, thioesterase, and CYP enzymes, can participate in the formation of 10-HDA or related trans-2 intermediates [6]. However, this heterologous evidence demonstrates biochemical feasibility in engineered microorganisms and does not prove that acyl-CoA dehydrogenase is the native enzyme responsible for double-bond formation in honeybee mandibular glands. A recent honeybee mandibular-gland study implicated d11ds/LOC551527, an acyl-CoA Δ11 desaturase-like homolog, as a candidate gene associated with the unsaturation step in 10-HDA biosynthesis. RNAi-mediated knockdown of d11ds significantly reduced 10-HDA levels, supporting its functional involvement, although direct enzymatic characterization of the native mandibular-gland reaction remains necessary [4]. Therefore, in this review, acyl-CoA dehydrogenase is described as a β-oxidation-associated enzyme or as a component identified in heterologous microbial systems, rather than as the confirmed native enzyme responsible for double-bond formation in honeybee mandibular glands. By contrast, d11ds/LOC551527 is presented as the currently supported honeybee mandibular-gland candidate associated with the unsaturation step in 10-HDA biosynthesis. Nevertheless, whether d11ds directly catalyzes trans double-bond formation on the native mandibular-gland substrate remains to be confirmed by enzymatic assays, substrate-feeding experiments, and subcellular localization studies. Figure 1 shows a schematic diagram of the 10-HDA biosynthetic pathway in the mandibular glands of honeybee workers.

## 3. Biological Activities of 10-HDA

As the quintessential unsaturated fatty acid in RJ, 10-HDA exhibits a multifaceted pharmacological profile. Emerging evidence has shifted the research paradigm from broad characterization to the precise elucidation of its molecular interactomes, especially concerning metabolic homeostasis, immunomodulatory signaling, and anti-inflammatory cascades. Beyond its established antimicrobial properties, 10-HDA has gained prominence for its antitumor potential, where it modulates key cellular pathways to inhibit malignancy. Moreover, 10-HDA confers significant therapeutic benefits in dermatological health, notably by accelerating wound healing and promoting tissue regeneration through the stimulation of collagen synthesis and fibroblast proliferation.

### 3.1. Anti-Inflammatory and Immunomodulatory Mechanisms

The anti-inflammatory and immunomodulatory efficacy of 10-HDA is orchestrated through a multi-layered regulatory network, encompassing the suppression of upstream signal transduction, the modulation of tissue-specific inflammasomes, and the metabolic reprogramming of immune cells. In cell-based studies, 10-HDA has been shown to attenuate pro-inflammatory signaling by interfering with TLR-related pathways. In RAW 264.7 macrophages, 10-HDA reduced LTA-induced NO, IL-6, TNF-α, and inflammatory-gene expression and inhibited MAPK phosphorylation [11]. In vascular smooth muscle cells, 10-HDA has been reported to interact with TLR4/MD-2 and reduce downstream NF-κB/MAPK activation; however, this evidence remains primarily cellular, lacking definitive in vivo proof of TLR4 inhibition [12]. Beyond systemic signaling, 10-HDA exerts profound homeostatic control across diverse physiological axes. In neuroinflammatory contexts, 10-HDA triggers the forkhead box protein–autophagy axis in microglia, which subsequently clears protein aggregates and inhibits nod-like receptor protein 3 (NLRP3) inflammasome assembly, thereby shielding neurons from lipopolysaccharide-induced apoptosis [13]. This protective role extends to the gut–liver axis, where 10-HDA reinforces the intestinal mucosal barrier and reshapes the microbiota to mitigate endotoxemia. Specifically, in colitis-associated liver damage, it prevents hepatocyte pyroptosis by blocking the TLR9-mediated assembly of the NLRP1 and NLRP3 inflammasomes [14,15,16]. Nevertheless, the translational relevance of these gut–liver axis findings should be interpreted cautiously. Current evidence is derived mainly from zebrafish, DSS-treated mice, and LPS-challenged chickens, with experimental exposures including 1–10 mg/L in adult zebrafish, 100 mg/kg/day in mice, and 1–5 g/kg in chicken diets [14,15,16]. Species-specific differences in immune signaling, microbiota composition, barrier physiology, disease models, routes of administration, and achievable human exposures mean that efficacy and safety cannot be directly extrapolated to human inflammatory or liver diseases without pharmacokinetic, dose–response, and clinical validation studies. At the systems level, integrative omics analyses underscore the capacity of 10-HDA to remodel arginine, proline, and glutathione metabolism, thereby effectively reversing immunometabolic dysregulation and restoring cellular homeostasis [17].

### 3.2. Metabolic Regulation and Bioenergetic Optimization

10-HDA exerts a profound influence on systemic glucose and lipid homeostasis while concurrently optimizing cellular bioenergetics, with mechanisms ranging from organ-system coordination to the fine-tuning of organelle function. Available evidence, derived mainly from reviews and preclinical models rather than clinical trials of isolated 10-HDA, suggests that royal jelly and its bioactive constituents may influence glucose and lipid metabolism under metabolic-disorder-related conditions [18]. Thus, 10-HDA is better viewed at present as a preclinical metabolic regulator with translational potential, while its effective dose range, pharmacokinetics, safety, and clinical relevance in metabolic syndrome remain to be established. At the subcellular level, research underscores the capacity of 10-HDA to stabilize the mitochondrial membrane potential and reinforce the transmembrane proton gradient. Such bioenergetic refinement enhances the efficiency of the coupled electron transport chain and facilitates ATP synthesis. Intriguingly, this metabolic upregulation is coupled with elevated NADPH production, which collectively fortifies cellular redox buffers and sustains the energetic demands of high-performance physiological processes [19]. These findings should, however, be interpreted in relation to the experimental exposure levels: the mitochondrial assays were performed mainly in UVA-stressed human skin fibroblasts treated with 0.25 mg/mL 10-HDA for 24 h, and the 3D epidermal model used 0.625 mg/mL 10-HAD [19]. Whether similar tissue concentrations can be achieved through dietary, oral, or topical use remains uncertain; therefore, the bioenergetic effects should be regarded as concentration-dependent in vitro observations pending validation of bioavailability. The regulatory scope of 10-HDA extends beyond generalized energy balance to include the precise maintenance of the anabolic–catabolic equilibrium within specialized tissues. In the pathophysiology of osteoarthritis, 10-HDA has been identified to specifically bind the C-terminal domain of aspartyl β-hydroxylase (ASPH). By perturbing ASPH-mediated downstream cascades, 10-HDA effectively thwarts the metabolic shifts associated with cellular senescence, thereby preserving chondrocyte function [20]. Together, these cell, tissue-explant, and animal-model findings support 10-HDA as a mechanistically interesting modulator of metabolic resilience but not yet as a clinically validated intervention for metabolic disease.

### 3.3. Antimicrobial Potency and Nano-Enabled Applications

Mechanistic studies have shown that 10-HDA can inhibit bacterial growth by disrupting membrane permeability and integrity. In *Escherichia coli* CICC23657, 10-HDA was tested over a concentration range of 0.625–10.0 mg/mL using the Oxford cup diffusion method, and a final concentration of 1.0 mg/mL was used in growth-curve and membrane-permeability assays. Under these conditions, 10-HDA delayed bacterial growth and promoted the leakage of K^+^, Ca^2+^, Mg^2+^, and 260 nm absorbing intracellular materials, suggesting membrane damage as a major antibacterial mechanism [21]. The antibacterial activity of 10-HDA is also influenced by environmental pH. Against *Paenibacillus larvae*, MIC values varied among strains and pH conditions; at pH 6.6, reported MICs ranged from approximately 0.60 to 2.80 μg/μL, whereas at pH 5.5, the MIC decreased to 0.20 μg/μL in the tested strains, indicating stronger inhibition under acidic conditions [22]. This pH dependence has practical implications for both royal jelly preservation and colony health. Because 10-HDA is a weak organic acid, its antibacterial activity may be enhanced in acidic matrices, helping to explain why the naturally acidic environment of royal jelly and larval food can contribute to microbial control. In apicultural practice, maintaining product acidity and avoiding storage conditions that raise pH may be relevant to preserving antibacterial activity, whereas in the hive, larval food and midgut pH may influence the extent to which 10-HDA restricts *Paenibacillus larvae* propagation. These implications remain context-dependent because strain susceptibility, spore versus vegetative state, and the complex royal jelly matrix can all modify the observed effect [22].

In addition, one study evaluating Anatolian royal jelly reported antibacterial activity against *Mycobacterium smegmatis* ATCC 607, with an MIC of 7.81 μg/mL for the royal jelly sample; however, this result should be interpreted with caution because the activity was measured for whole royal jelly rather than for purified 10-HDA alone [23]. Recent nanoformulation studies further suggest that delivery systems may enhance the antibacterial performance of 10-HDA. For *Staphylococcus aureus* ATCC 6538, free 10-HDA showed an MIC of 50 μg/mL and an MBC of 800 μg/mL, whereas 10-HDA-functionalized carbon dots reduced the MIC and MBC to 25 μg/mL and 200 μg/mL, respectively. At 400 μg/mL, 10-HDA inhibited *S. aureus* biofilm formation by 54.5 ± 9.26%, while 10-HDA-functionalized carbon dots achieved 91.2 ± 0.66% inhibition [24]. Nevertheless, the hydrogel/carbon-dot work should be presented as a single proof-of-concept delivery study rather than as definitive evidence that nano-enabled 10-HDA formulations will broadly outperform free 10-HDA across pathogens or clinical settings. Its results are valuable for illustrating how formulation can alter local concentration, release, and biofilm activity in a *Staphylococcus aureus* wound model, but broader significance requires replication across bacterial species, wound types, safety endpoints, and formulation designs [24]. These findings indicate that 10-HDA has measurable antibacterial activity in vitro, but its efficacy varies with bacterial species, pH, formulation strategy, and whether the test material is purified 10-HDA or a complex royal jelly-derived matrix.

### 3.4. Antitumor Potential and Epigenetic Remodeling

Preclinical evidence suggests that the antitumor potential of 10-HDA and royal jelly-derived preparations may involve epigenetic remodeling, programmed cell death, oxidative-stress regulation, and modulation of inflammatory signaling. At the epigenetic level, royal jelly treatment, rather than purified 10-HDA alone, has been reported to affect DNA methylation-related endpoints by suppressing DNA methyltransferase activity and shifting selected tumor suppressor gene (TSG) promoters toward a more transcriptionally permissive state in cervical, colorectal, and lung carcinoma cell models. The reactivation of these silenced TSGs subsequently antagonizes downstream oncogenic programs, including aberrant angiogenesis, dysregulated lipid metabolism, and hyperactive protein synthesis [25]. In an Ehrlich solid tumor mouse model, 10-HDA was associated with increased pro-apoptotic markers (Bax, Caspase-3), reduced Bcl-2 expression, and improved oxidative stress indices, including lower lipid peroxidation and nitric oxide levels, along with higher SOD, CAT, and GPx activity. Furthermore, in preclinical tumor models, 10-HDA has been reported to enhance the antitumor effects of cyclophosphamide when used in combination, suggesting a possible chemosensitizing effect [26]. However, this observation should be interpreted cautiously because it is based on experimental models, and further pharmacokinetic, safety, and clinical studies are needed before therapeutic relevance can be established. Within the TME, cell-based evidence from WiDr human colon cancer cells indicates that 10-HDA can reduce NF-κB signaling and alter the secretion of inflammatory mediators, including IL-8, IL-1β, TNF-α, and IL-1ra, suggesting a potential immunomodulatory contribution to antitumor activity [27].

Despite these mechanistic signals, the current evidence for antitumor activity remains preliminary. The cited studies are based mainly on cancer cell lines, a mouse Ehrlich solid tumor model, or whole royal jelly preparations, and no clinical studies of purified 10-HDA as an anticancer intervention are available in this section. Bioavailability is also unresolved: 10-HDA is a fatty acid with formulation- and matrix-dependent absorption, and the concentrations used in cell models, including millimolar-range exposures in WiDr cells, may not correspond to achievable tumor tissue levels after oral intake. Pharmacokinetic parameters such as absorption, distribution, metabolism, clearance, tumor accumulation, and interaction with chemotherapeutic agents, therefore, require systematic evaluation. Safety considerations are equally important; although short-term mouse studies reported no obvious changes in liver or kidney enzyme levels at 2.5–5 mg/kg/day, this does not establish long-term safety, the maximum tolerated dose, reproductive safety, immunological risk, or safety in combination with chemotherapy. Consequently, 10-HDA should be described as a preclinical antitumor lead rather than a clinically validated anticancer therapy.

### 3.5. Dermatological Applications and Cutaneous Regeneration

10-HDA exerts dual functionality in dermatology by directly modulating cellular repair circuitry and acting as a synergistic bio-template within advanced biomaterials to facilitate accelerated tissue regeneration. In the domain of anti-aging and dermal structural preservation, 10-HDA attenuates collagen degradation by suppressing the expression of matrix metalloproteinases, particularly those involved in UV-induced photoaging. Complementing its structural benefits, 10-HDA exhibits potent tyrosinase-inhibitory activity, effectively downregulating melanogenesis and positioning this lipid as a promising natural candidate for skin-lightening therapeutics [28]. However, these dermatological applications should be interpreted with caution because most available evidence derives from in vitro cell models, reconstructed epidermal models, or animal wound models rather than human topical trials. As an unsaturated fatty acid intended for cutaneous use, 10-HDA may also require systematic evaluation of local tolerance, including potential irritation, barrier disruption, sensitization, and photoreactivity, particularly when applied to inflamed or damaged skin. The efficacy of 10-HDA in epidermal homeostasis and wound resolution is further characterized by its activation of the peroxisome proliferator-activated receptor alpha signaling axis. This ligand-mediated activation upregulates the synthesis of critical barrier proteins, including filaggrin, thereby expediting the assembly of the cornified envelope and reinforcing the skin’s permeability barrier [19,24]. Notably, the cited epidermal and fibroblast experiments used defined model-system concentrations, such as 0.25–0.625 mg/mL 10-HDA, so translation to topical products depends on whether comparable amounts can reach the viable epidermal or dermal layers without compromising tolerability [20]. When encapsulated within nanostructured delivery systems, 10-HDA significantly promotes the closure of recalcitrant and infected wounds. This underscores a sophisticated synergy between its intrinsic pharmacological potency and bioengineering strategies in driving cutaneous recovery [19,24]. At the formulation level, delivery systems may improve stability, local retention, and controlled release, but they also introduce practical challenges related to 10-HDA hydrophobicity, loading efficiency, release kinetics, penetration depth, batch reproducibility, and the safety assessment of carrier materials [24,28]. Accordingly, 10-HDA is best described as a promising preclinical dermatological lead, while clinically relevant dosing, formulation stability, skin delivery, and topical tolerability remain unresolved.

The key experimental contexts and evidence levels discussed in Section 3 are summarized in Table 1.

## 4. Anatomy and Physiological Functions of the Honeybee Mandibular Gland

### 4.1. Structural Organization of the Mandibular Gland in Worker Bees

**Figure 2:**
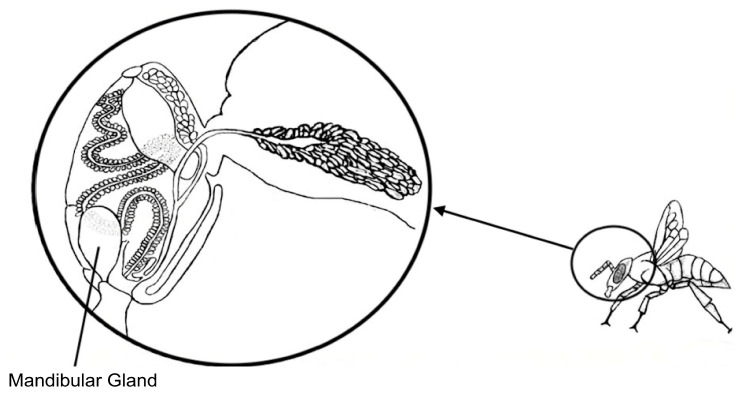
Structure of the mandibular glands in bees [34].

The mandibular glands of the honeybee are a pair of prominent, sac-like exocrine organs situated bilaterally within the head capsule. These glands exhibit pronounced caste dimorphism, reaching their maximal development in queens, followed by workers, while remaining vestigial in drones [9,29,30]. Histologically, these organs are classified as Class III insect epidermal glands, characterized by a voluminous central reservoir surrounded by dense clusters of secretory units. Each unit functions as a bicellular complex, comprising a hypertrophied secretory cell and a specialized duct cell. The proximal terminus of the duct cell invaginates into the secretory cell to form the end apparatus—a dedicated organelle for secretion collection—while the distal segment converges into the central lumen, ultimately discharging through an orifice at the mandibular base [31,32,33]. Throughout the honeybee’s life cycle, the glandular architecture undergoes dynamic remodeling—a phenomenon governed by age-related polyethism. This plasticity ensures that the rate of 10-HDA production aligns with the shifting social roles of the worker bee, from nurse bee secretion to forager-level maintenance [29,33]. The structure of the mandibular glands in bees is shown in Figure 2.

### 4.2. Physiological Versatility and Functional Plasticity of the Mandibular Gland

The mandibular glands of worker bees exhibit extraordinary phenotypic plasticity, characterized by adaptive remodeling of their developmental state and secretory profiles in synchrony with age-related polyethism and colony demands. During the nursing phase, the glands are markedly hypertrophied and dedicated to vigorous lipid metabolism, primarily synthesizing 10-HDA and its precursor, 10-HDAA [30,35]. Integrative omics profiles have confirmed robust upregulation of β-oxidation machinery during this window, facilitating high-titer flux of 10-HDA. Beyond its nutritional and antimicrobial roles, 10-HDA functions as a potent histone deacetylase inhibitor. In this capacity, it exerts epigenetic governance over larval caste fate by modulating developmental trajectories toward either the queen or worker phenotype [10,36]. As worker bees transition from nursing to foraging or defensive roles, the biosynthetic machinery undergoes a tactical shift toward the production of volatile methyl ketones, predominantly 2-heptanone [32]. Within this behavioral context, 2-heptanone serves as a multifunctional chemical mediator. As an alarm pheromone, it orchestrates the recruitment of nestmates for collective defense. During agonistic interactions with intruders, such as *Varroa destructor* or wax moth larvae, 2-heptanone acts as a localized anesthetic; when delivered via mandibular incisions, it paralyzes the adversary to enhance defensive efficacy [37]. Furthermore, foragers deploy 2-heptanone as a “forage-marking pheromone” to tag depleted floral resources, thereby preventing redundant foraging efforts and optimizing the colony’s collective energetic efficiency [10,38]. Under acute social perturbations, such as the loss of a queen, the mandibular glands exhibit profound functional reversion. In “laying workers”, the typical age-associated glandular atrophy is arrested; instead, these glands undergo a physiological metamorphosis to acquire queen-like traits. The secretory chemotype undergoes a qualitative transition toward the synthesis of queen mandibular pheromones (QMP) [39,40]. This pheromonal mimicry allows laying workers to suppress the ovarian development of their nestmates, thereby asserting reproductive dominance within the queenless hierarchy [39].

The mandibular gland of queens is highly specialized for the production of QMP, which is dominated by 9-oxo-2-decenoic acid (9-ODA) and 9-hydroxy-2-decenoic acid. Its biosynthesis is associated with the differential expression of key enzymes, including aldehyde dehydrogenase 1 and medium-chain acyl-CoA dehydrogenase [41]. Functionally, QMP maintains colony stability by regulating worker reproduction, behavior, endocrine status, and retinue response, while 9-ODA also acts as a long-range sex pheromone that attracts drones during mating flights [42,43,44,45,46,47,48,49]. In contrast, the drone mandibular gland is structurally reduced and functionally limited compared with those of queens and workers. It lacks the developed biosynthetic machinery required for high-level fatty acid production, and proteomic evidence indicates the absence of key enzymes involved in lipid β-oxidation and 10-HDA biosynthesis [9,10,41]. Nevertheless, its secretions may function as volatile aggregation pheromones, helping recruit drones to aggregation areas during nuptial flights [30,49].

## 5. Regulatory Factors and Mechanisms Governing 10-HDA Biosynthesis and Secretion

### 5.1. Developmental Maturation and Ontogeny

The 10-HDA secretory capacity of worker bee mandibular glands varies with developmental ontogeny and age-related polyethism. Available studies indicate pronounced temporal dynamics, from low 10-HDA levels in newly emerged bees to a high-output phase in nurse-stage workers. This pattern is associated with morphological remodeling, including gland enlargement and changes in organelle-rich secretory tissues, as well as transcriptomic and proteomic shifts. To avoid overstating causality, the evidence discussed below is distinguished as follows: age-, role-, or omics-based associations are treated as correlational evidence; RNAi or dietary perturbation studies are treated as functional validation; and direct enzyme assays, binding assays, or rescue experiments are required for definitive mechanistic proof.

Glandular maturation is strongly associated with 10-HDA biosynthetic capacity, although most age-series studies remain observational. From a morpho-physiological perspective, the mandibular glands of newly emerged worker bees are typically immature or weakly secretory, and 10-HDA yields are correspondingly low. During the transition into the nursing phase, these glands undergo hypertrophic maturation, and 10-HDA output increases markedly. This developmental trajectory has been reported in both *Apis cerana cerana* and *Apis mellifera ligustica*, although the precise peak age varies across studies and experimental conditions [50,51]. As workers shift toward foraging and defensive roles, mandibular gland activity and 10-HDA accumulation generally decline or become more variable. These patterns support a developmental association, but they do not by themselves prove that any single morphological feature causes the change in 10-HDA production.

At the molecular level, transcriptomic profiling has identified candidate pathways that covary with the developmental and behavioral transitions of the mandibular gland. The proposed 10-HDA biosynthetic trajectory includes fatty acid hydroxylation, successive β-oxidation cycles, and terminal desaturation or functional group modification. Genes encoding candidate enzymes in these steps, including *CYP6AS8*, *FAS*, *ETF-β*, *KAT*, and desaturase-family genes, often show stage- or condition-dependent expression patterns in nurse bees and foragers [52,53]. These expression data should be interpreted primarily as correlative evidence. Stronger functional validation is available for several targets: RNAi-mediated knockdown of CYP6AS8 reduces 10-HDA content in *Apis mellifera* workers, supporting its contribution to the hydroxylation-related step rather than fully elucidating its catalytic mechanism in vivo [54]. Similarly, RNAi targeting ETF-β or *KAT* reduces 10-HDA accumulation and alters mandibular gland morphology, supporting their involvement in β-oxidation-associated metabolism [55]. More recently, cross-species transcriptomic analysis and RNAi targeting an acyl-CoA Delta(11) desaturase gene (*d11ds*) showed a marked reduction in 10-HDA levels, while PPI and co-expression analyses identified Kay and Drep-2 as potential transcriptional regulators [4]. However, these regulatory links remain mechanistic inferences until direct evidence of binding, reporter activity, rescue, or enzyme activity is available.

Proteomic and metabolic profiling further support, but do not independently prove, a coordinated developmental program for 10-HDA production. High-output worker stages show enrichment of proteins involved in energy metabolism, protein folding, antioxidant capacity, and intracellular transport, including ATP synthase subunits, HSP-family chaperones, and Ran GTPase [50,51]. These proteins are biologically plausible contributors to the metabolic demand of fatty acid biosynthesis, but their current evidence level is mainly proteomic correlation unless targeted perturbation confirms effects on 10-HDA output. Taken together, the literature supports a tiered model in which developmental maturation sets the physiological context, candidate lipid-metabolic genes provide functional entry points, and the upstream regulatory network remains an important area for mechanistic verification. The levels of evidence regarding the developmental and ontogenetic regulation of 10-HDA biosynthesis are presented in Table 2.

### 5.2. Bee Species and Strains

Genetic background is an important determinant of mandibular-gland function, but its effects should be interpreted alongside worker age, behavioral role, and colony context. The available evidence in this subsection mainly compares high royal jelly-producing bees (RJBs) with Italian bees (ITBs, *Apis mellifera ligustica*), rather than providing a broad cross-species survey that includes Apis cerana. RJBs represent a selectively bred strain with markedly increased royal jelly yield; however, high yield should not be treated as a simple proxy for universally superior 10-HDA biosynthetic capacity or colony performance.

At the molecular level, phosphoproteomic data indicate that RJBs and ITBs share a core mandibular-gland phosphoproteome across newly emerged, nurse, and forager stages, while RJBs show a larger number of identified phosphoproteins at each stage (509, 554, and 460 in RJBs vs. 483, 499, and 387 in ITBs, respectively) [56]. The largest inter-strain divergence occurs in nurse bees, where RJBs display more complex phosphorylation patterns related to energy metabolism, fatty acid synthesis, protein processing, cytoskeletal organization, and intracellular signaling [56]. These findings support the view that long-term selection for royal jelly production has reshaped post-translational regulation in the mandibular gland. Nevertheless, they are primarily omics-level associations; several kinase–substrate relationships and protein functions were inferred from homology or bioinformatic prediction and still require targeted functional validation [56].

The selective-breeding context also introduces important limitations. RJBs can produce more than tenfold higher royal jelly yields than ITBs, but the same source notes that 10-HDA content in royal jelly may decline to some extent as yield increases [56]. Thus, selection for bulk royal jelly production may create a trade-off between yield and compositional quality. In addition, intensive selection for a narrow production trait may reduce genetic diversity, increase disease or stress susceptibility, or compromise colony-level fitness traits such as brood viability, overwintering ability, foraging performance, and resilience under nutritional stress. These concerns are not resolved by mandibular-gland omics alone and should be evaluated through colony-level, multi-generation breeding studies. Genetic background, strain selection, and evidence related to 10-HDA synthesis are shown in Table 3.

### 5.3. Extrinsic Environmental Factors

Extrinsic environmental factors modulate 10-HDA biosynthesis and secretion through nutritional supply, endocrine status, colony-management intensity, forage background, and environmental stress. Direct evidence is strongest for dietary fatty acids, carbohydrate sources, citric acid supplementation, juvenile hormone analog (JHA) exposure, botanical or pollen sources, queen-cell number, and pesticide-contaminated pollen. By contrast, the specific effects of climate change and non-pesticide environmental pollutants on 10-HDA remain less directly quantified and should be presented as important industry-relevant uncertainties.

Dietary nutrition is a major external modulator of 10-HDA biosynthesis. Oleic acid (OA) supplementation in *Apis mellifera ligustica* increased royal jelly 10-HDA from 1.36 ± 0.06% to 1.57 ± 0.07% after 60 d of colony feeding with 8% OA, and worker-head 10-HDA increased 1.13-fold and 1.20-fold at 9 and 18 d, respectively [57]. Exogenous stearic acid also increased 10-HDA in royal jelly and worker heads in Zhejiang royal jelly bees but reduced feed intake and transiently affected colony strength, indicating that fatty acid supplementation should be evaluated together with colony-level fitness indices [58].

Carbohydrate source, organic acids, and endocrine cues further shape royal jelly quality. In *Apis mellifera*, 10-HDA varied from 1.40% to 2.77% across sugar-feeding and regional treatments, with the highest value observed in glucose-fed colonies from *Doğanşehir* and the lowest in unfed controls from the same region [59]. Citric acid (CA) supplementation promoted gland development and improved royal jelly quality; 0.50% CA increased 10-HDA from 1.725 ± 0.005% to 1.995 ± 0.005%, whereas 0.75% CA strongly extended worker lifespan but did not produce the same 10-HDA maximum [60]. JHA effects appear age- and dose-dependent rather than uniformly beneficial; excessive or mistimed exposure can be adverse, so endocrine manipulation should be interpreted as mechanistically sensitive rather than as a simple production strategy [61].

Botanical and geographic origin, pollen diet, and production load have direct practical relevance. Regional differences in feeding experiments suggest that forage background can interact with nutrition, as mean 10-HDA differed between *Doğanşehir* (2.38 ± 0.64%) and *Uluköy* (2.07 ± 0.40%) [59]. More specifically, royal jelly from honeybees fed monofloral Acer mono pollen contained a higher average 10-HDA than that from bees fed *Phellodendron amurense* pollen; 10-HDA decreased soon after single-pollen feeding, peaked at 18–24 d, and declined again by day 30 [62]. Queen-cell number provides a management example: increasing the grafting load from 64 to 320 queen cells reduced 10-HDA from 2.01% to 1.52%, and five strips required 25% more grafting labor without a significant yield advantage over four strips [63]. Thus, pollen source, feeding duration, and larval load should be optimized together rather than considered separately.

Pesticide exposure now has more direct evidence for effects on royal jelly composition. In a colony-level study using a multi-pesticide pollen treatment, residues detected in royal jelly were low, yet exposed colonies produced royal jelly with significantly altered metabolomic, proteomic, and phytosterol profiles, including lower relative abundance of 10-HDA and major royal jelly proteins [64]. This suggests that pesticide-contaminated food can impair nurse-bee secretory output through indirect physiological pathways even when chemical transfer into royal jelly is limited. Climate change and non-pesticide pollution, including heat stress, drought, altered flowering phenology, heavy metals, and air- or soil-derived pollutants, remain plausible but under-quantified risks for 10-HDA production; future studies should pair residue or stress measurements with 10-HDA assays, gland development, and colony fitness endpoints.

Microbial synthetic biology studies should also be interpreted cautiously in relation to honeybee physiology. Engineered microbial systems can produce 10-HDA at titers of approximately 217–486.5 mg/L and are useful for industrial biomanufacturing and for generating hypotheses about precursor supply and enzymatic requirements [65]. Nevertheless, these systems do not demonstrate that the same pathway operates in the honeybee mandibular gland; any connection to in vivo 10-HDA synthesis should be explicitly framed as hypothesis-generating rather than mechanistic proof. The external environmental factors affecting 10-HDA synthesis and royal jelly quality are shown in Table 4.

## 6. Conclusions

The biosynthesis and secretion of 10-HDA represent a sophisticated biochemical process involving substrate conversion, mandibular gland maturation, and multi-tiered genetic, nutritional, endocrine, and environmental regulation. Existing studies have established a useful metabolic blueprint, including the conversion of long-chain fatty acid precursors toward 10-HDA and the involvement of fatty acid metabolism, β-oxidation, hydroxylation, gland development, and colony-management factors in determining RJ quality.

However, several uncertainties and unresolved controversies remain. First, although the stearic-acid-to-10-HDA route has been proposed, the relative contribution of alternative precursors, the identity of the true rate-limiting enzymes, and the compartmental organization of β-oxidation and terminal hydroxylation in mandibular gland cells are not yet fully resolved. Second, the regulatory hierarchy among genotype, worker age, nutrition, endocrine status, botanical source, and production load remains difficult to disentangle because many studies differ in colony background, sampling time, analytical method, and management conditions. Third, the biological activities attributed to 10-HDA should be interpreted cautiously: purified 10-HDA, whole RJ, RJ-derived matrices, and nanoformulations are not equivalent experimental systems, and reported effects often depend on purity, dose, exposure time, model organism, and delivery strategy. At present, most pharmacological evidence is derived from in vitro or preclinical models, while pharmacokinetic behavior, bioavailability, long-term safety, effective physiological dose ranges, and clinical relevance remain insufficiently established.

Future work should therefore move beyond technological expansion alone and address these conceptual gaps directly. High-resolution spatiotemporal mapping of mandibular glands, supported by single-cell transcriptomics, spatial metabolomics, isotope tracing, and targeted genetic or enzymatic validation, will be needed to define the cellular sites and rate-limiting steps of 10-HDA synthesis. Standardized experimental designs should also be adopted to compare bee subspecies, nutrition, floral sources, endocrine cues, and apicultural load under controlled conditions. For biological activity studies, greater attention should be paid to 10-HDA form and purity, dose justification, exposure time, formulation effects, pharmacokinetics, and safety endpoints. Finally, synthetic biology and microbial biomanufacturing remain promising routes for scalable 10-HDA production, but their products must be benchmarked against natural RJ-derived 10-HDA in terms of chemical equivalence, impurity profile, bioactivity, regulatory acceptability, and practical cost-effectiveness.

## Figures and Tables

**Table 1:** Evidence map of reported biological activities of 10-HDA.

Model System	10-HDA Form/Purity	Dose	Exposure Time	Key Outcomes	Evidence Level
RAW 264.7 macrophages; LTA-induced mouse lung injury [11]	10-HDA; Sigma; purity NR	Cells: 1–5 mM; mice: 100 mg/kg	Cells: 1 h pre + 24 h LTA; mice: 7 d pre	Lower NO/cytokines and MAPK/NF-κB signaling; reduced lung injury	Cell + animal
Mouse VSMCs stimulated with Ang II or LPS [12]	10-HDA; ≥98%	1–4 mM; main dose 4 mM	1 h pre + 6 h stimulus	Blocked TLR4-related signaling; reduced cytokines/ROS; increased IL-10/GSH/SOD	Cell mechanism
LPS neuroinflammation in mice and BV-2 cells [13]	10-HDA; >99%	Mice: 100 mg/kg/day; BV-2:1–4 μM	Mice: 30 d; cells: 18–24 h	Reduced glial activation and cytokines; activated FOXO1-autophagy; inhibited NF-κB/NLRP3	Animal + cell
IMI-induced liver injury in zebrafish larvae/adults [14]	10-HDA; 98%	Larvae: 0.5–1 mM; adults: 1–10 mg/L	Larvae: 48 h; adults: 14 d	Reduced liver injury, oxidative stress, inflammation and gut dysbiosis	Zebrafish in vivo
DSS colitis-associated liver injury in mice [15]	10-HDA; >99%	100 mg/kg/day	7 d DSS period; day 8 sampling	Reduced ALT/AST, cytokines and TLR9-NLRP1/NLRP3 pyroptosis markers	Animal
LPS-challenged chickens [16]	Dietary 10-HDA; ≥98%	1 or 5 g/kg diet; LPS 0.5 mg/kg	Diet d1–21; LPS d17/19/21	Improved barrier, cytokines, antioxidant status and microbiota	Animal
LPS-stimulated RAW 264.7 omics model [17]	10-HDA; Sigma; purity NR	10 μg/mL + LPS 1 μg/mL	2 h pre + 24 h LPS	Reprogrammed inflammatory metabolites/genes, especially amino acid and lipid pathways	Cell omics
RJ/metabolic-disorder literature review [18]	RJ/constituents; not purified 10-HDA	Heterogeneous; review only	NA	Suggests metabolic and GI relevance; purified 10-HDA evidence remains limited	Review
EpiKutis, HaCaT and HSF skin models [18]	Biosynthesized 10-HDA; purity NR	0.625 mg/mL or 0.25 mg/mL	24 h	Activated PPARα/barrier genes; improved lipid and mitochondrial endpoints	Skin models
Chondrocytes, human OA explants and DMM mice [20]	10-HDA; purity NR	Cells/explants: 2–10 nM; mice: 100 mg/kg/day	Cells: 48 h; explants: 7 d; mice: 7 weeks	Protected cartilage, reduced pain behavior and identified ASPH as a target	Cell + ex vivo + animal
*E. coli* CICC23657 membrane-damage assays [21]	10-HDA; >99%	0.625–10 mg/mL screen; 1 mg/mL assays	24 h; leakage at 3/5/7 h	Delayed growth and caused ion/260-nm material leakage	Antibacterial cell assay
*Paenibacillus larvae* strains under different pH [22]	10-HDA; purity NR	MIC: 0.20–2.80 μg/μL	MIC time NR	pH-dependent inhibition; stronger at acidic pH; spores more sensitive	Antibacterial assay
Anatolian RJ against *M. smegmatis* [23]	Whole RJ; not purified 10-HDA	RJ MIC: 7.81 μg/mL	Culture ~24 h; MIC time NR	RJ inhibited *M. smegmatis*; attribution to 10-HDA alone uncertain	Matrix-level
*S. aureus*, biofilm and infected-wound hydrogel models [24]	10-HDA ≥ 97%; free, CDs, hydrogel	50–1600 μg/mL; biofilm 400 μg/mL; hydrogel 1 mg/g	In vitro 24 h; wounds to day 12	10-HDA@CDs improved antibacterial/biofilm effects and accelerated wound healing	Biomaterial + animal
HeLa, HT29 and A549 treated with RJ [25]	Whole RJ; not purified 10-HDA	RJ: 100 μg/mL^−1^ g/mL; DNMT assay 100 mg/mL	DNMT: 120 min; cells: 72 h	RJ inhibited DNMT activity and altered methylation/gene-expression profiles	RJ cell evidence
Ehrlich solid tumor mice [26]	10-HDA; >99%	2.5 or 5 mg/kg; ± CP 25 mg/kg	Oral daily for 2 weeks	Reduced tumor markers/oxidative stress; increased apoptosis markers; enhanced CP response	Animal
WiDr colon cells and bacterial pathogens [27]	RJ-purified 10-HDA; ~92%	Cells: 0.1–5 mM; MIC: 23–44 μM or 40–43 μM	Cells: 24 h; antibacterial time NR	Reduced inflammatory cytokines/NF-κB; increased IL-1ra; inhibited multiple pathogens	Cell + antibacterial
RJDS release, NHDF cells and skin tape stripping [28]	RJDS matrix; 10-HDA encapsulation > 85%	RJDS: 0.01–0.1% *v*/*v*; ~1142 ppm 10-HDA	Cells: 24 h; release/stability to 24th week	Stabilized/released 10-HDA; promoted NHDF growth and skin-regeneration genes	Formulation + exploratory human

NR, not reported in the supplied full text; NA, not applicable.

**Table 2:** Evidence levels for developmental and ontogenetic regulation of 10-HDA biosynthesis.

Evidence Focus	Species	Model/Design	Key Outcome	Evidence Level	Refs.
Age-related 10-HDA titer	*Apis cerana cerana*	0–55 d worker age series; HPLC and proteomics	10-HDA is low in newly emerged workers, peaks around the nursing stage, and then declines; FAS is higher in the high-output stage.	Correlation	[52]
Age-related morphology and titer	*Apis mellifera ligustica*	0–30 d worker age series; HPLC/GC-MS, MG dissection, SEM	10-HDA rises from day 0 to a later high-output stage; mature glands are larger and more structured than newly emerged glands.	Correlation	[51]
Gene expression across age/diet	*Apis mellifera ligustica*	qPCR age series and diet comparison	FAS and related genes covary with 10-HDA trends, but expression patterns alone do not prove causality.	Correlation	[52]
Transcriptome by role/nutrition	*Apis mellifera*	RNA-seq/qPCR; age, role, and nutrition contrasts	Candidate lipid-metabolic genes, including CYP6AS8 and β-oxidation genes, are associated with MG secretory status and nutritional condition.	Correlation/condition association	[53]
CYP6AS8 function	*Apis mellifera ligustica*	Caged workers; dsRNA feeding; qPCR and GC quantification	CYP6AS8 knockdown significantly reduces 10-HDA content, supporting involvement in hydroxylation-related biosynthesis.	Functional validation	[54]
ETF-β and KAT function	*Apis mellifera ligustica*	Age expression, precursor feeding, and dsRNA injection	ETF-β/KAT expression follows 10-HDA trends; RNAi reduces 10-HDA and alters MG morphology, supporting β-oxidation involvement.	Functional validation	[55]
d11ds and candidate regulation	*Apis mellifera*; *Apis cerana*	NEB/NB/FB RNA-seq, WGCNA, cross-species comparison, RNAi	d11ds knockdown reduces 10-HDA by >50%; Kay and Drep-2 are proposed upstream regulators from co-expression/PPI evidence.	Functional validation; mechanistic inference	[4]
Proteomic support proteins	*Apis mellifera ligustica*	0 d vs. 25 d MG proteomics; 2-DE and MALDI-TOF/TOF	ATP synthase, HSP-family proteins, Ran, and other metabolic/transport proteins are enriched in high-output glands.	Proteomic correlation	[51]
Proteomic maturation markers	*Apis cerana cerana*	0 d vs. 10 d MG comparative proteomics	Differential proteins include lipid metabolism, protein folding, and energy-production categories linked to high 10-HDA stages.	Proteomic correlation	[50]

Correlation = age-, role-, transcriptomic-, proteomic-, or metabolite-level association; functional validation = targeted perturbation such as RNAi or precursor feeding that changes 10-HDA output; mechanistic proof = direct biochemical or regulatory demonstration, which remains limited for several proposed links. NEB, newly emerged bee; NB, nurse bee; FB, forager bee.

**Table 3:** Genetic background, strain selection, and evidence related to 10-HDA synthesis.

Evidence Focus	Species	Design	Quantitative Comparison	Main Interpretation	Refs.
Selective breeding outcome	RJB vs. ITB	Strain comparison summarized in phosphoproteomic thesis	RJB royal jelly yield >10-fold higher than ITB; 10-HDA content declined to some extent as yield increased	Selection improves bulk RJ yield, but yield and 10-HDA concentration may trade off	[56]
MG phosphoprotein number	RJB vs. ITB	LC-MS/MS phosphoproteomics across NE, N, and F stages	RJB: 509/554/460 phosphoproteins; ITB: 483/499/387 at NE/N/F	RJB MGs show a more extensive phosphorylation network at all stages	[56]
Nurse-stage strain effect	RJB vs. ITB	Stage-specific phosphoproteome comparison	Largest divergence occurs in the N stage; RJB has the highest phosphoprotein, peptide, and site counts	Nurse-stage phosphorylation is a likely regulatory layer supporting high RJ output	[56]
Functional categories in RJB MG	RJB vs. ITB	Bioinformatic pathway and protein-network analysis	Key categories: energy metabolism, fatty acid synthesis, protein processing, cytoskeleton, signaling	These categories plausibly support MG secretion, but many links are inferred rather than experimentally proven	[56]

RJB, high royal jelly-producing bee; ITB, Italian bee; MG, mandibular gland; NE, newly emerged bee; N, nurse bee; F, forager bee; d, day.

**Table 4:** Extrinsic environmental factors affecting 10-HDA synthesis and royal jelly quality.

Factor	Species Studied	Experimental Conditions	Magnitude of Effect	Practical Significance	Refs.
Oleic acid (OA)	*Apis mellifera ligustica*	Caged bees fed 2–8% OA; colonies fed 8% OA for 60 d	RJ 10-HDA: 1.57 ± 0.07% vs. 1.36 ± 0.06%; worker-head 10-HDA increased 1.13-fold (9 d) and 1.20-fold (18 d)	Promising fatty acid supplement, but colony-scale validation is needed	[57]
Stearic acid (SA)	Zhejiang royal jelly bees	10 colonies; 2% SA supplementation for 75 d	RJ and worker-head 10-HDA increased (*p* < 0.05); feed intake decreased; day-24 colony strength reduced	May improve quality, but colony fitness and feeding response must be monitored	[58]
Sugar source and region	*Apis mellifera*	Two regions; control, glucose, sucrose, and bee-feed treatments	10-HDA range: 1.40–2.77%; highest in Doğanşehir-glucose (2.77%), lowest in Doğanşehir-control (1.41%)	Carbohydrate management should be adapted to local forage background	[59]
Citric acid (CA)	*Apis mellifera*	Laboratory and colony feeding with 0–0.75% CA	0.50% CA increased RJ 10-HDA to 1.995 ± 0.005% vs. 1.725 ± 0.005%; 0.75% CA extended lifespan by 64.73%	0.50% CA appears most favorable for 10-HDA; higher dose may favor longevity instead	[60]
JHA exposure	*Apis mellifera ligustica*	Topical JHA treatments, including 25, 50, and 200 μg	50 μg promoted 10-HDA secretion in 3-day-old workers; high or mistimed exposure increased adverse effects	Endocrine effects are dose- and age-sensitive; not a simple field intervention	[61]
Bee pollen botanical source	Honeybees	Six colonies; monofloral Acer mono (82%) or Phellodendron amurense (86%) pollen; RJ collected after 6, 12, 18, 24, and 30 d feeding	10-HDA decreased initially, peaked at 18–24 d, then declined by 30 d; average 10-HDA was higher in RJ-Am than in RJ-Pa	Pollen source and feeding duration should be managed; forced single-pollen diets may reduce nutritional balance	[62]
Botanical/geographic origin	*Apis mellifera*	Regional and feeding comparisons	Mean 10-HDA: Doğanşehir 2.38 ± 0.64% vs. Uluköy 2.07 ± 0.40%	Apiary location and forage planning may affect RJ quality	[59]
Production load (queen-cell number)	Royal jelly bees	64, 128, 192, 256, and 320 queen cells per colony	10-HDA declined from 2.01% to 1.52%; 5 strips required 25% more grafting labor without yield benefit over 4 strips	Avoid over-grafting; four strips/256 cells were recommended under the tested system	[63]
Pesticide-contaminated pollen	*Apis mellifera* L.	Six colonies; treated pollen contained 9 added pesticides plus contaminants for 46 d; three grafting rounds	RJ residues were low (≤7 ppb except thymol), but 10-HDA differed significantly (W = 225, *p* < 0.01); RJ mass averaged 198 ± 6.6 mg vs. 283 ± 6.2 mg in controls, not significant	Pesticides can impair RJ nutritional composition indirectly even when residues in RJ are minimal	[64]
Microbial synthetic biology	Engineered microbes, not bees	Whole-cell or engineered microbial production routes	Reported titers about 217–486.5 mg/L in microbial systems	Industrial production model; only hypothesis-generating for honeybee physiology	[65]

## Data Availability

No new data or processed data were created in this paper. Data sharing is not available for this paper.

## Abbreviations

RJ	royal jelly
10-HDA	10-hydroxy-2-decenoic acid
FAS	fatty acid synthase
CYP450	cytochrome P450
10-HDAA	10-hydroxydecanoic acid
TLR4	Toll-like receptor 4
MD2	myeloid differentiation protein-2
NF-κB	nuclear factor-κB
NLRP3	nod-like receptor protein 3
ASPH	aspartyl β-hydroxylase
TME	tumor microenvironment
TSG	tumor suppressor gene
QMP	queen mandibular pheromones
9-ODA	9-oxo-2-decenoic acid
ALDH1	aldehyde dehydrogenase 1
JH	juvenile hormone
DAAs	drone aggregation areas
KAT	3 -ketoacyl- CoA thiolase
ETF-β	electron transfer flavoprotein β
RJBs	royal jelly bees
Am	*Apis mellifer* *a*
Ac	*Apis cerana*
MRJPs	major royal jelly proteins
JHAs	juvenile hormone analogues
CA	citric acid
OA	oleic acid
SA	stearic acid
